# Supplementary material for: Natural Plasmodium falciparum Infection Stimulates Human Antibodies to MSP1 Epitopes Identified in Mice Infection Models upon Non-Natural Modified Peptidomimetic Vaccination
Source: Molecules. 2023 Mar 10;28(6):2527. doi: 10.3390/molecules28062527 (PMC10057838; doi:10.3390/molecules28062527)
Supplement: Supplementary file 1 [file molecules-28-02527-s001.zip › Suplementary Figures.pptx]

## Slide 1
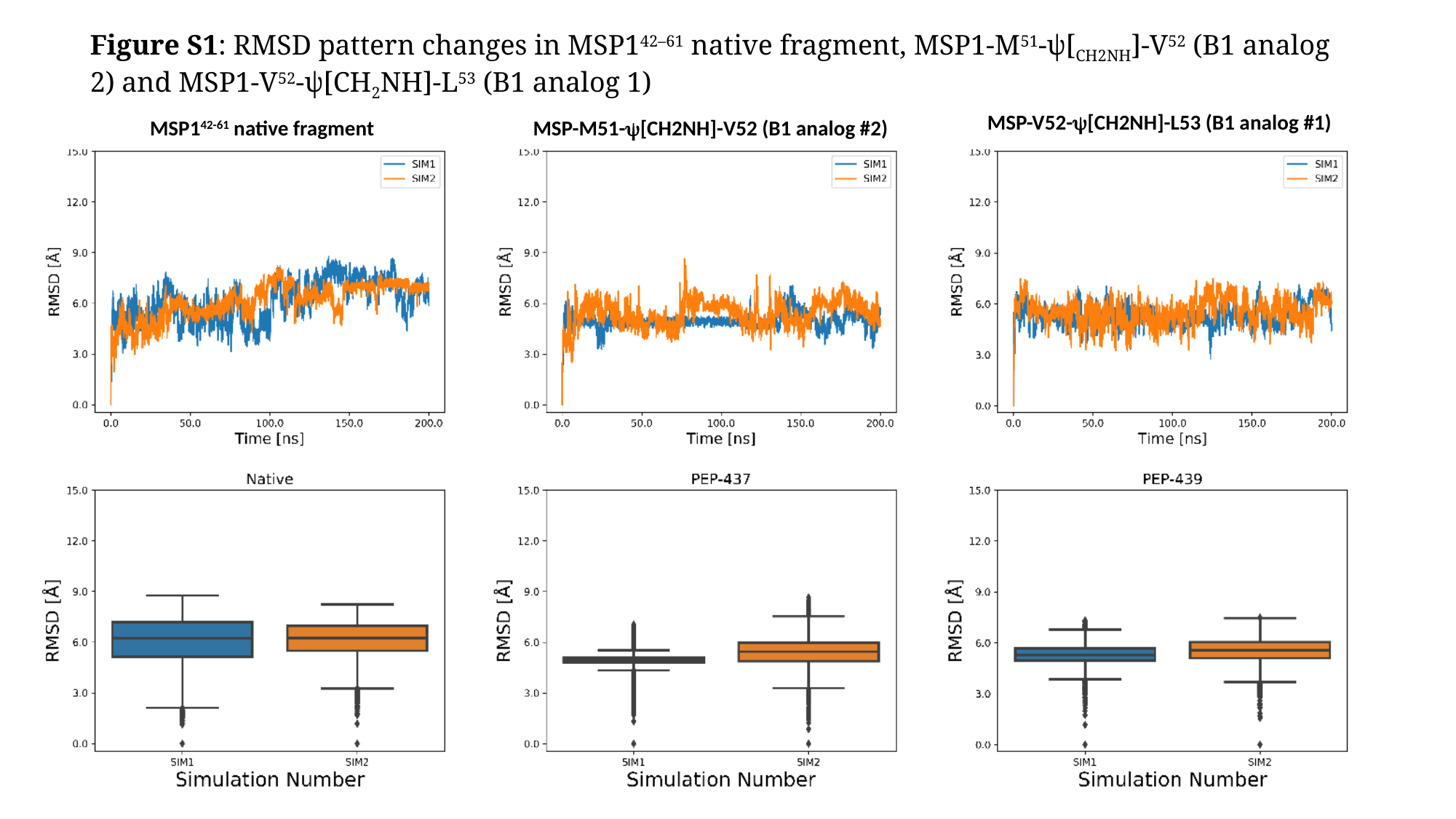

Figure S1: RMSD pattern changes in MSP142–61 native fragment, MSP1-M51-ψ[CH2NH]-V52 (B1 analog 2) and MSP1-V52-ψ[CH2NH]-L53 (B1 analog 1)
MSP-V52-[CH2NH]-L53 (B1 analog #1)
MSP142-61 native fragment
MSP-M51-[CH2NH]-V52 (B1 analog #2)

## Slide 2
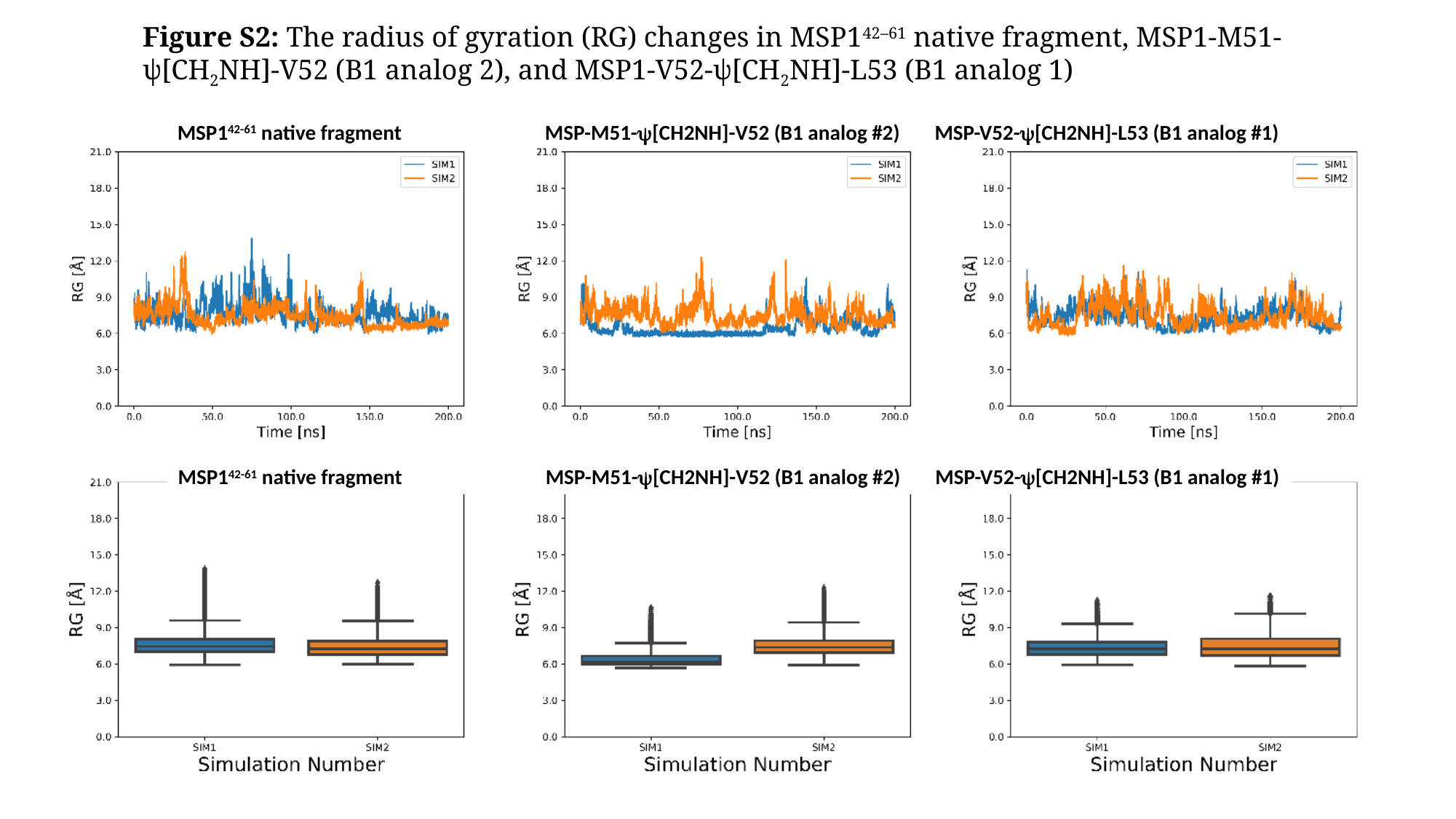

Figure S2: The radius of gyration (RG) changes in MSP142–61 native fragment, MSP1-M51-ψ[CH2NH]-V52 (B1 analog 2), and MSP1-V52-ψ[CH2NH]-L53 (B1 analog 1)
MSP142-61 native fragment
MSP-M51-[CH2NH]-V52 (B1 analog #2)
MSP-V52-[CH2NH]-L53 (B1 analog #1)
MSP142-61 native fragment
MSP-M51-[CH2NH]-V52 (B1 analog #2)
MSP-V52-[CH2NH]-L53 (B1 analog #1)

## Slide 3
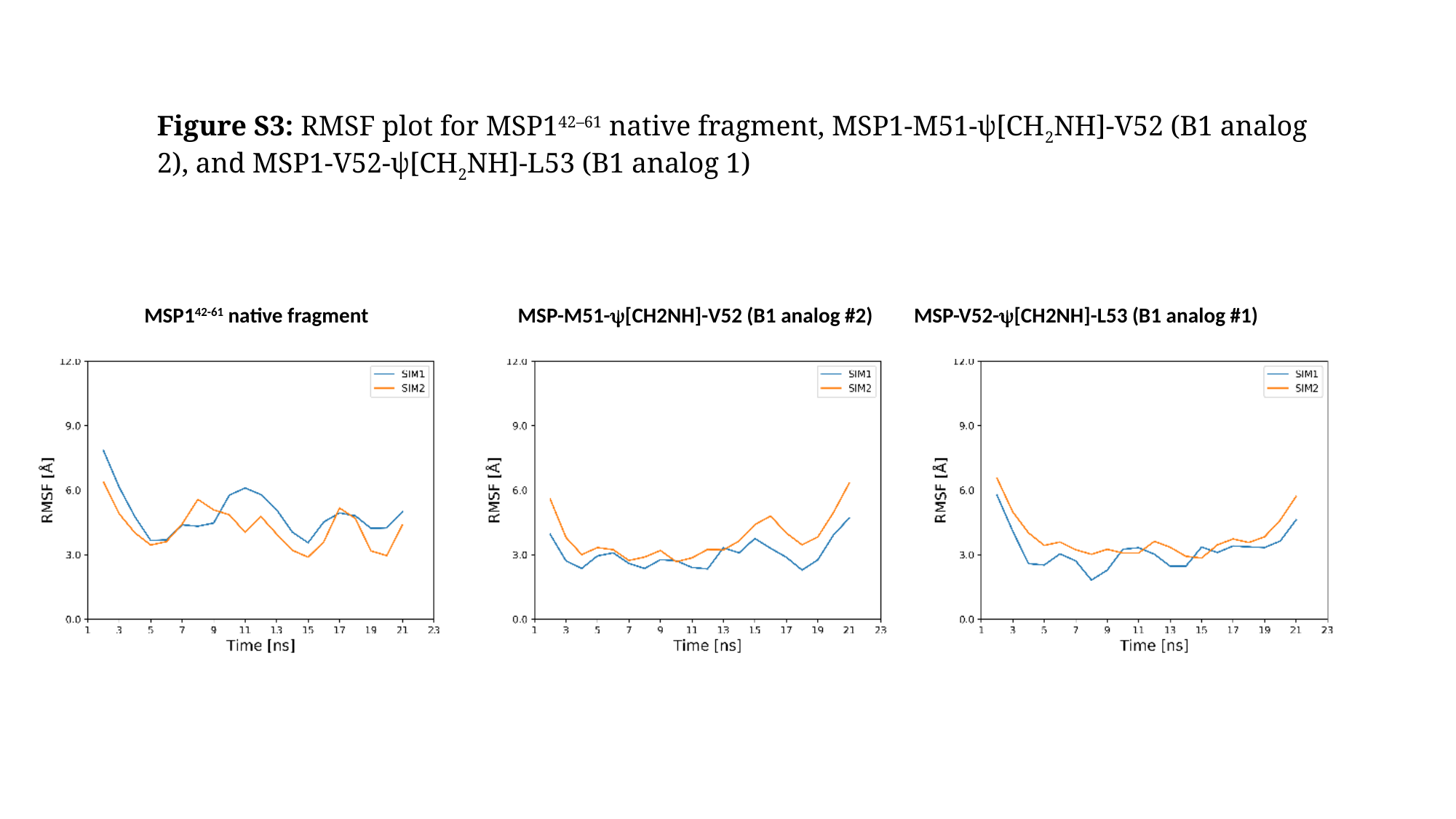

Figure S3: RMSF plot for MSP142–61 native fragment, MSP1-M51-ψ[CH2NH]-V52 (B1 analog 2), and MSP1-V52-ψ[CH2NH]-L53 (B1 analog 1)
MSP142-61 native fragment
MSP-M51-[CH2NH]-V52 (B1 analog #2)
MSP-V52-[CH2NH]-L53 (B1 analog #1)

## Slide 4
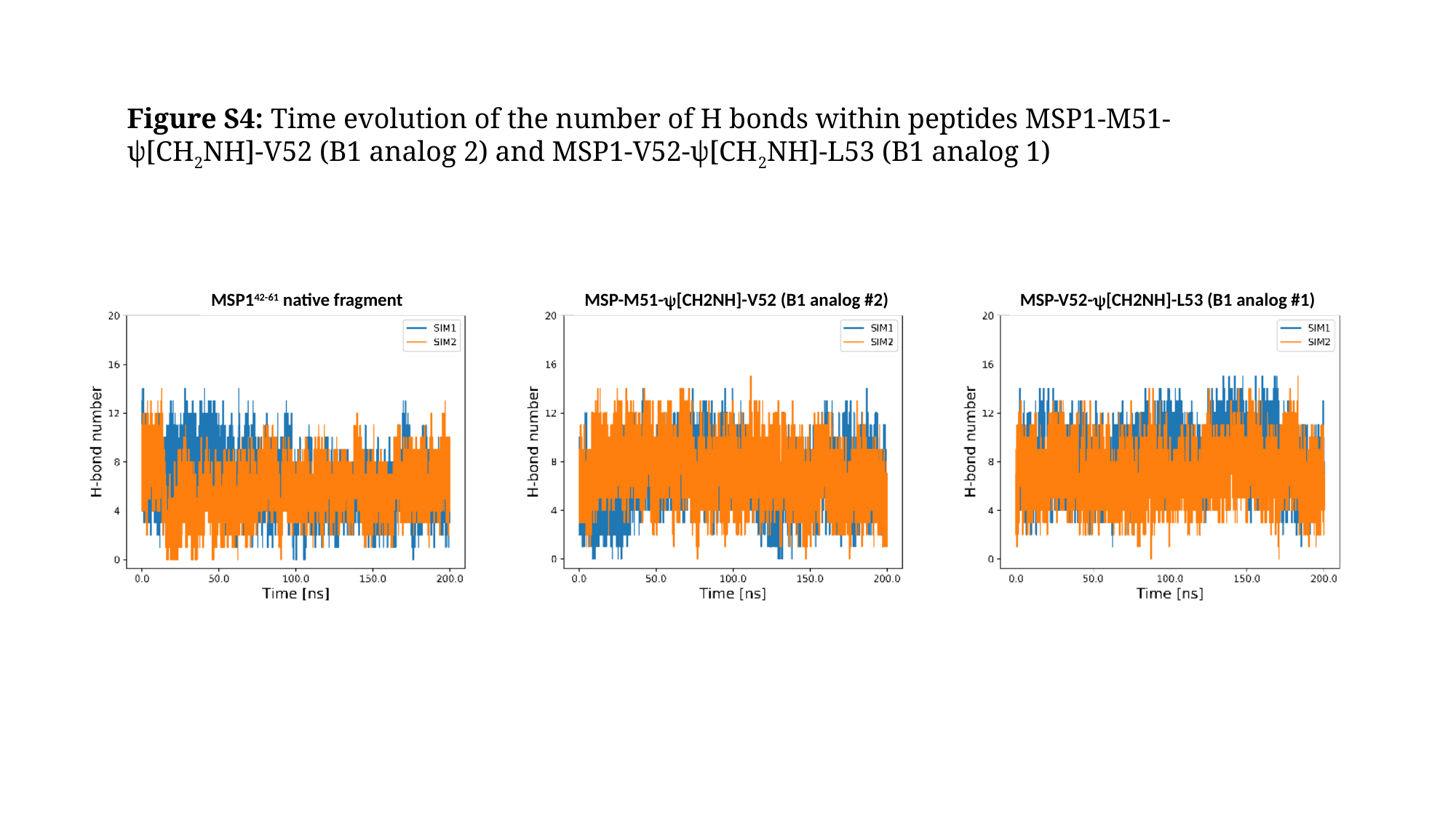

Figure S4: Time evolution of the number of H bonds within peptides MSP1-M51-ψ[CH2NH]-V52 (B1 analog 2) and MSP1-V52-ψ[CH2NH]-L53 (B1 analog 1)
MSP142-61 native fragment
MSP-M51-[CH2NH]-V52 (B1 analog #2)
MSP-V52-[CH2NH]-L53 (B1 analog #1)

## Slide 5
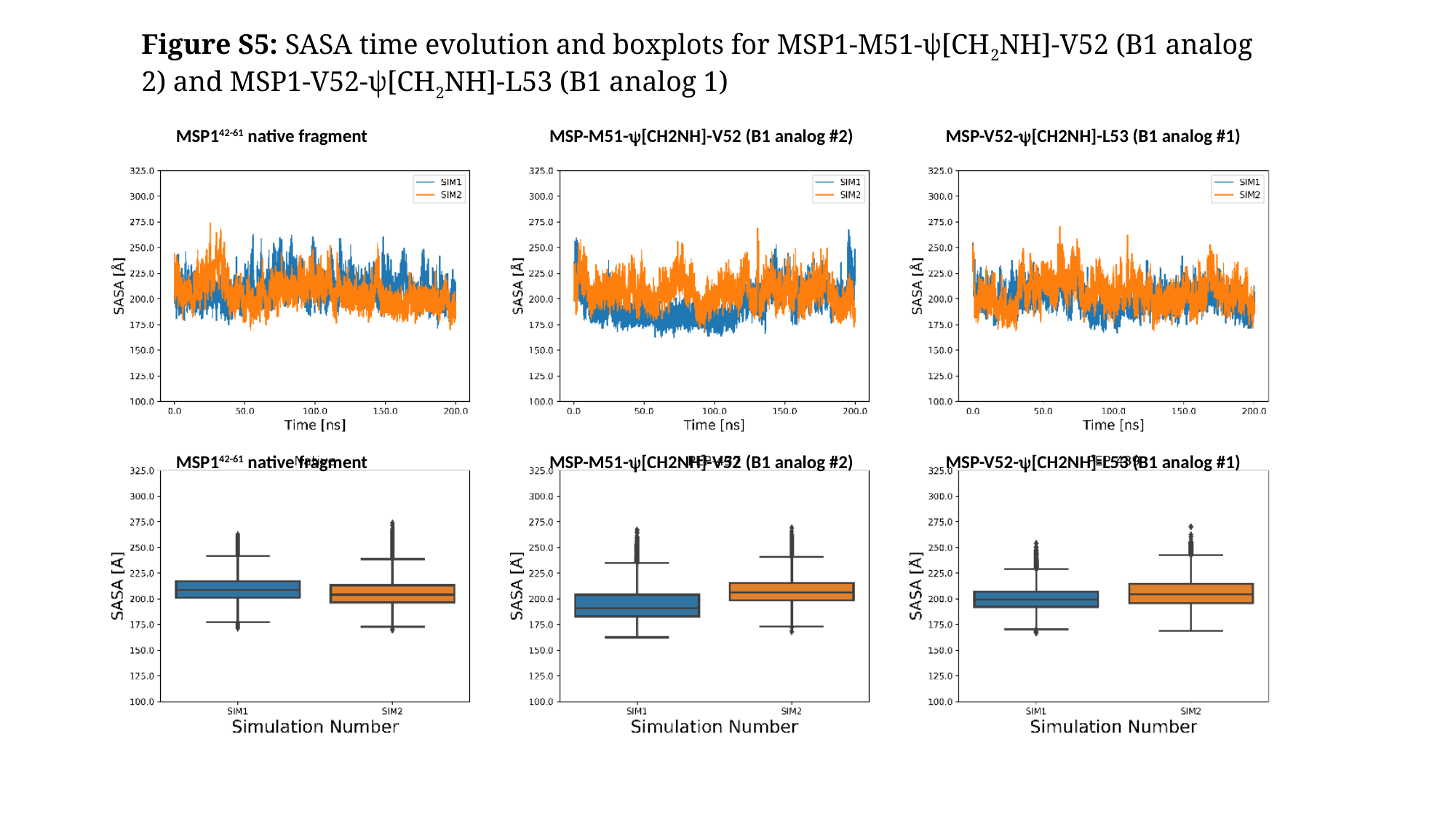

Figure S5: SASA time evolution and boxplots for MSP1-M51-ψ[CH2NH]-V52 (B1 analog 2) and MSP1-V52-ψ[CH2NH]-L53 (B1 analog 1)
MSP142-61 native fragment
MSP-M51-[CH2NH]-V52 (B1 analog #2)
MSP-V52-[CH2NH]-L53 (B1 analog #1)
MSP142-61 native fragment
MSP-M51-[CH2NH]-V52 (B1 analog #2)
MSP-V52-[CH2NH]-L53 (B1 analog #1)

## Slide 6
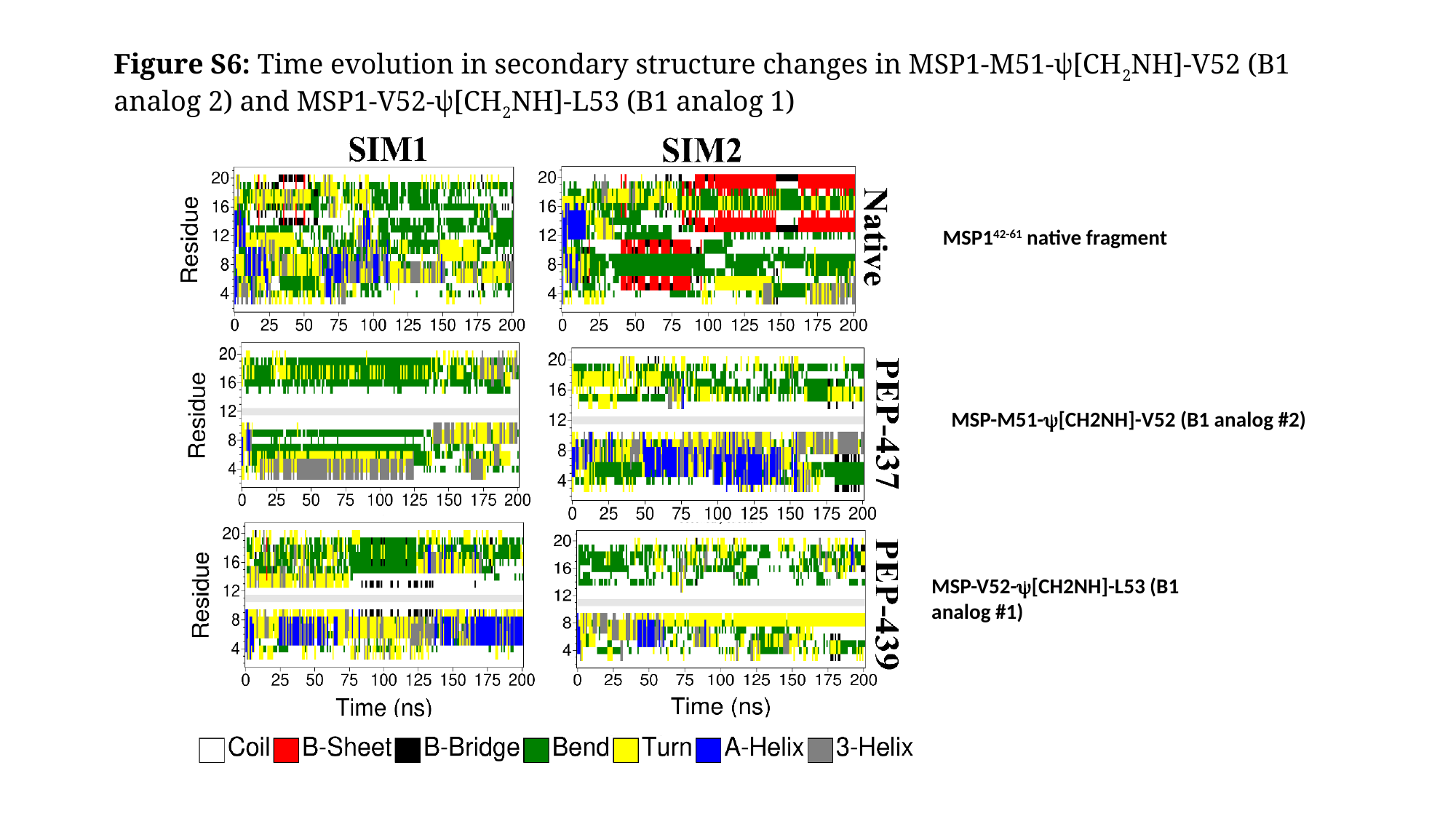

Figure S6: Time evolution in secondary structure changes in MSP1-M51-ψ[CH2NH]-V52 (B1 analog 2) and MSP1-V52-ψ[CH2NH]-L53 (B1 analog 1)
MSP142-61 native fragment
MSP-V52-[CH2NH]-L53 (B1 analog #1)
MSP-M51-[CH2NH]-V52 (B1 analog #2)

## Slide 7
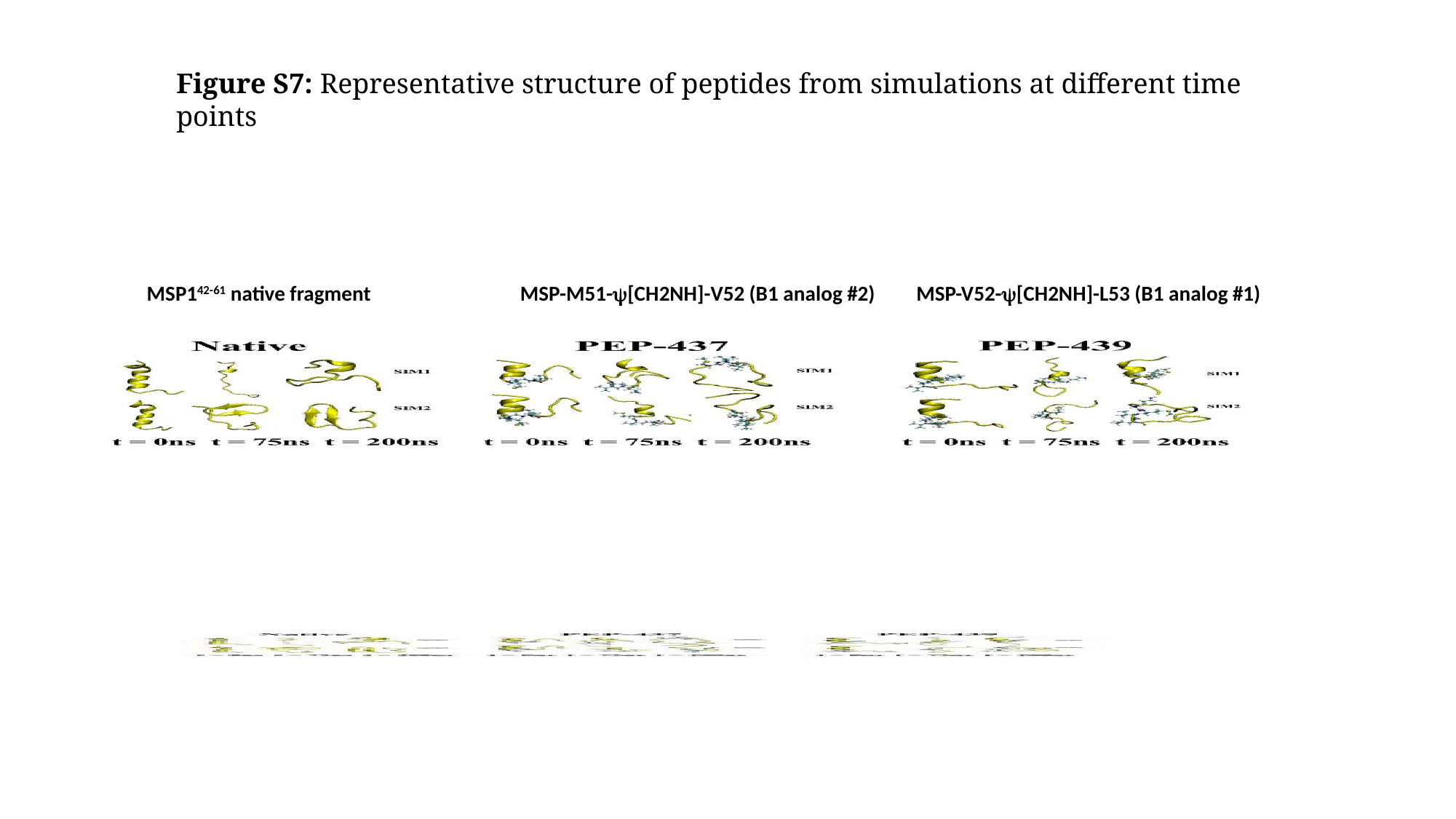

Figure S7: Representative structure of peptides from simulations at different time points
MSP142-61 native fragment
MSP-M51-[CH2NH]-V52 (B1 analog #2)
MSP-V52-[CH2NH]-L53 (B1 analog #1)
