# Supplementary figures and images for: Natural Plasmodium falciparum Infection Stimulates Human Antibodies to MSP1 Epitopes Identified in Mice Infection Models upon Non-Natural Modified Peptidomimetic Vaccination
Source: Molecules. 2023 Mar 10;28(6):2527. doi: 10.3390/molecules28062527 (PMC10057838; doi:10.3390/molecules28062527)

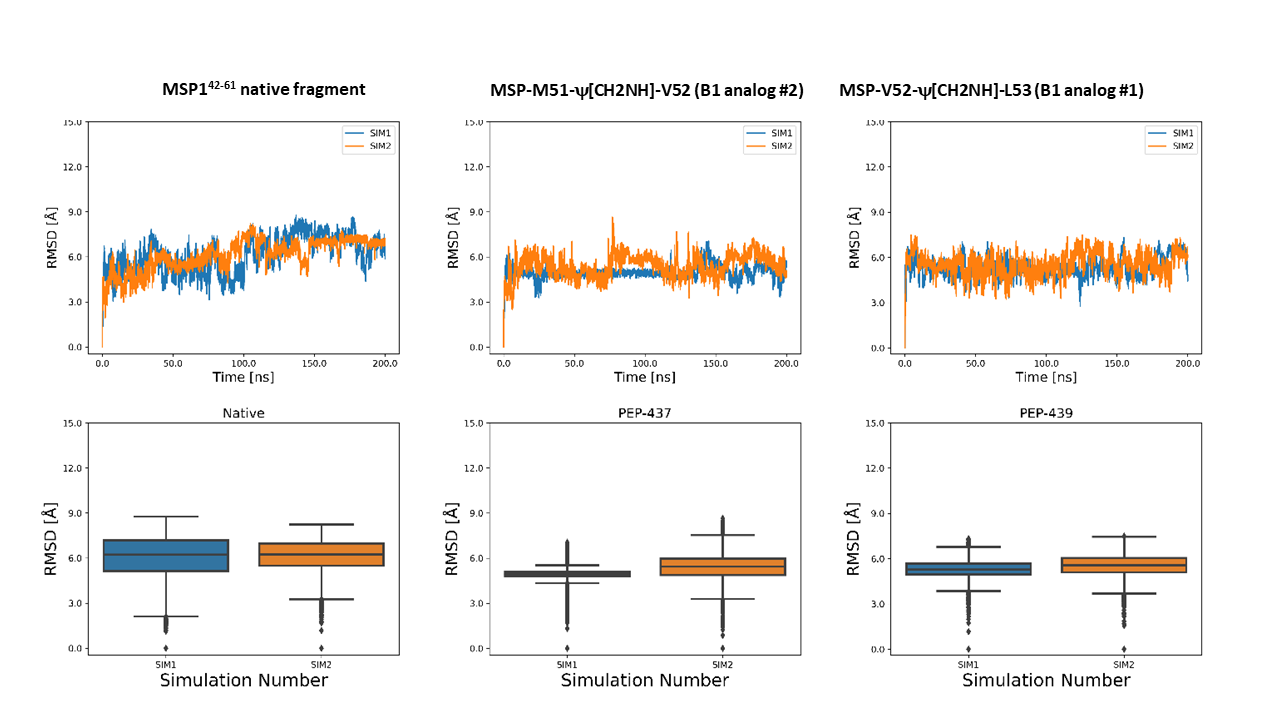

Supplement: Supplementary file 1 [file molecules-28-02527-s001.zip › Figure S1.TIF]

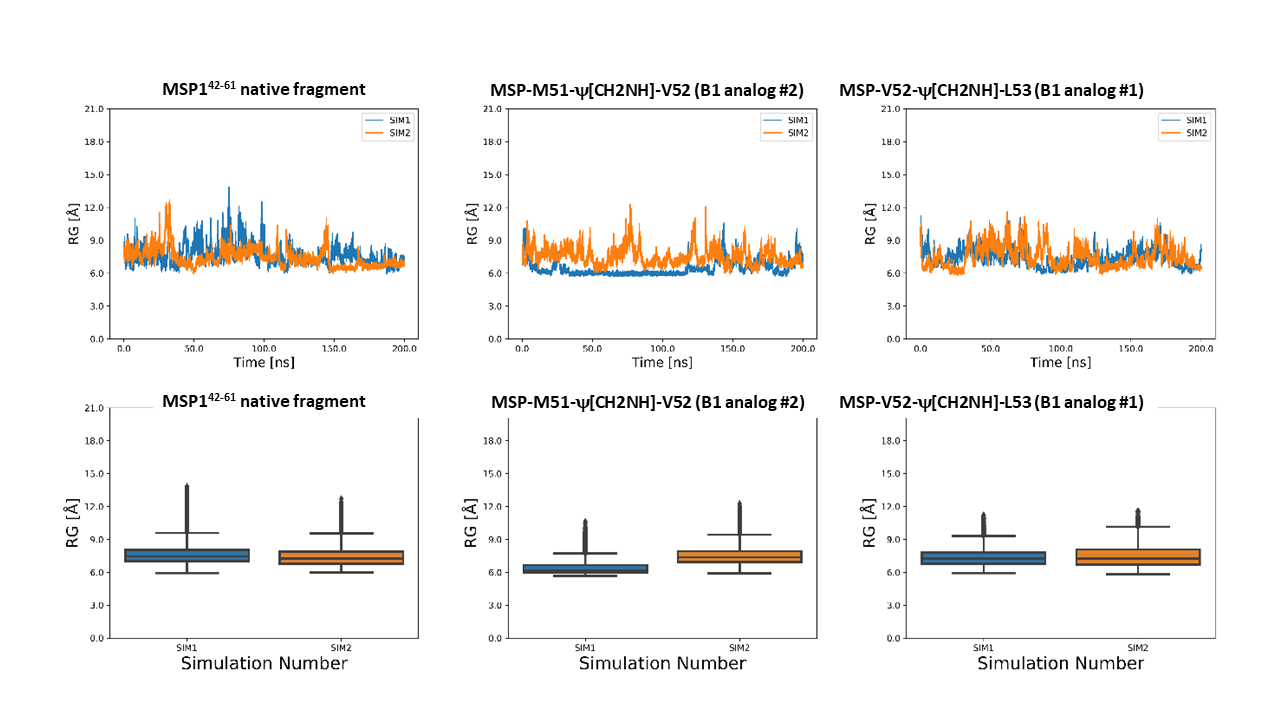

Supplement: Supplementary file 1 [file molecules-28-02527-s001.zip › Figure S2.TIF]

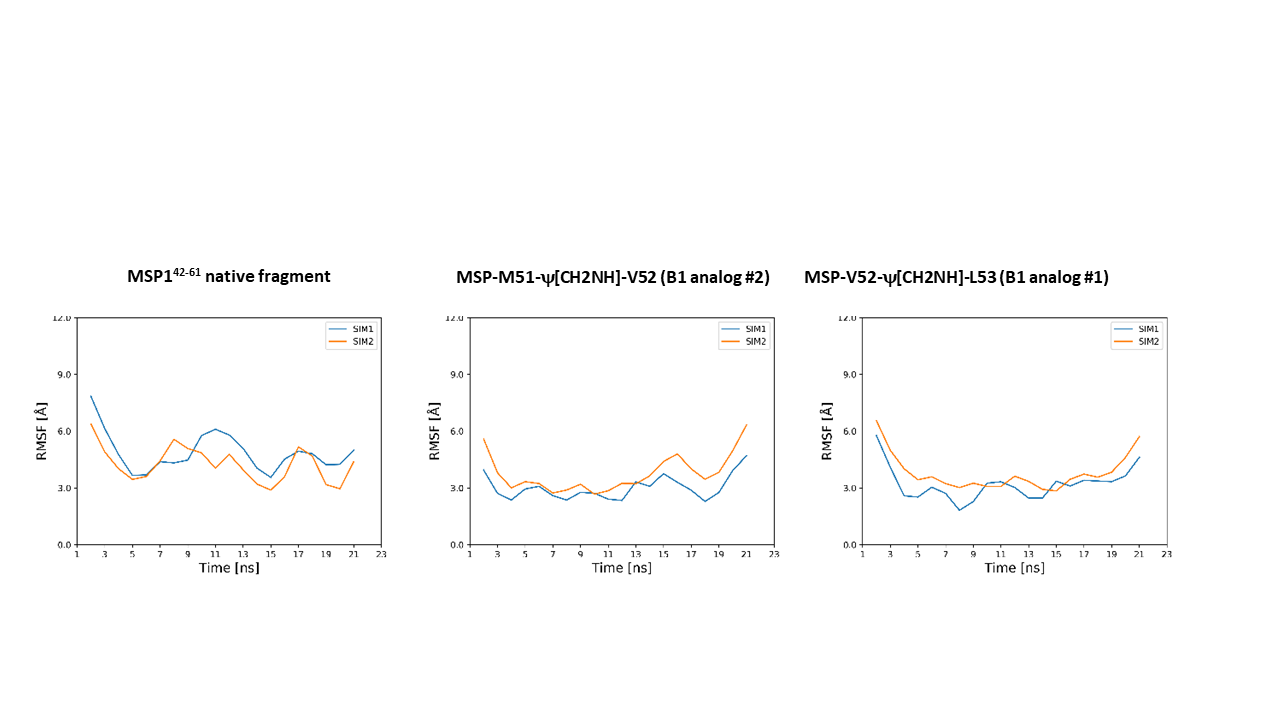

Supplement: Supplementary file 1 [file molecules-28-02527-s001.zip › Figure S3.TIF]

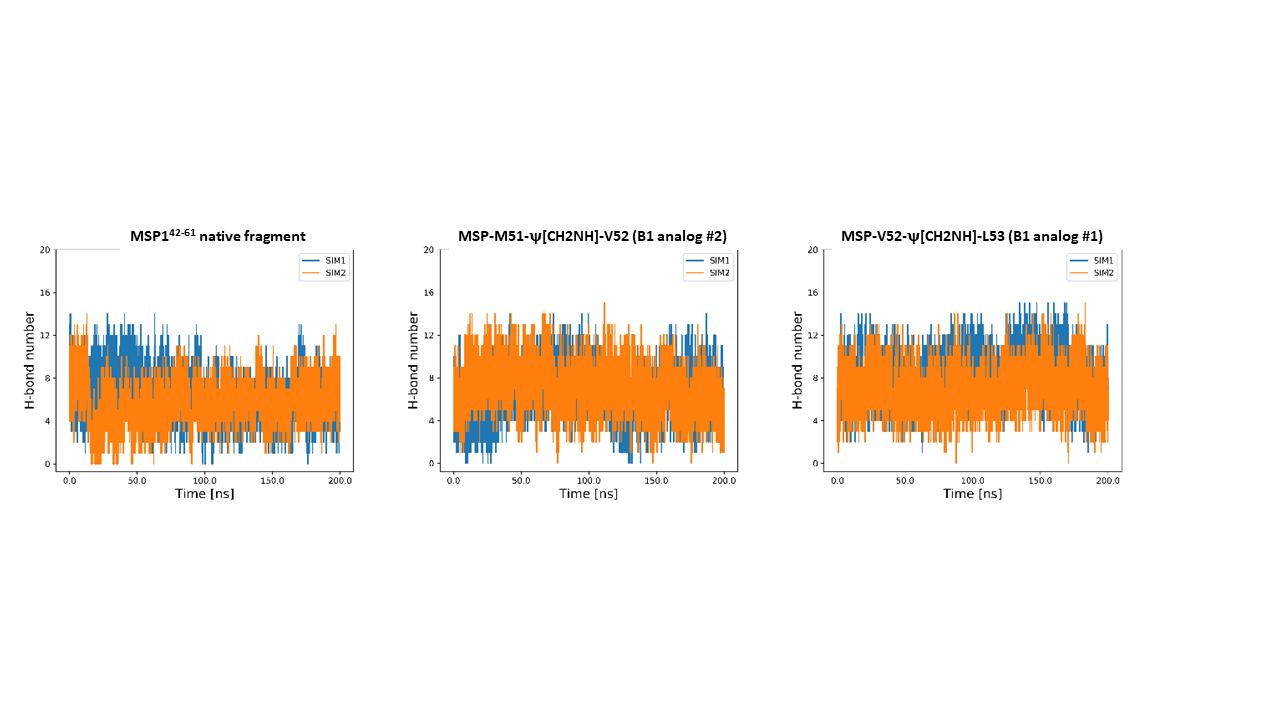

Supplement: Supplementary file 1 [file molecules-28-02527-s001.zip › Figure S4.TIF]

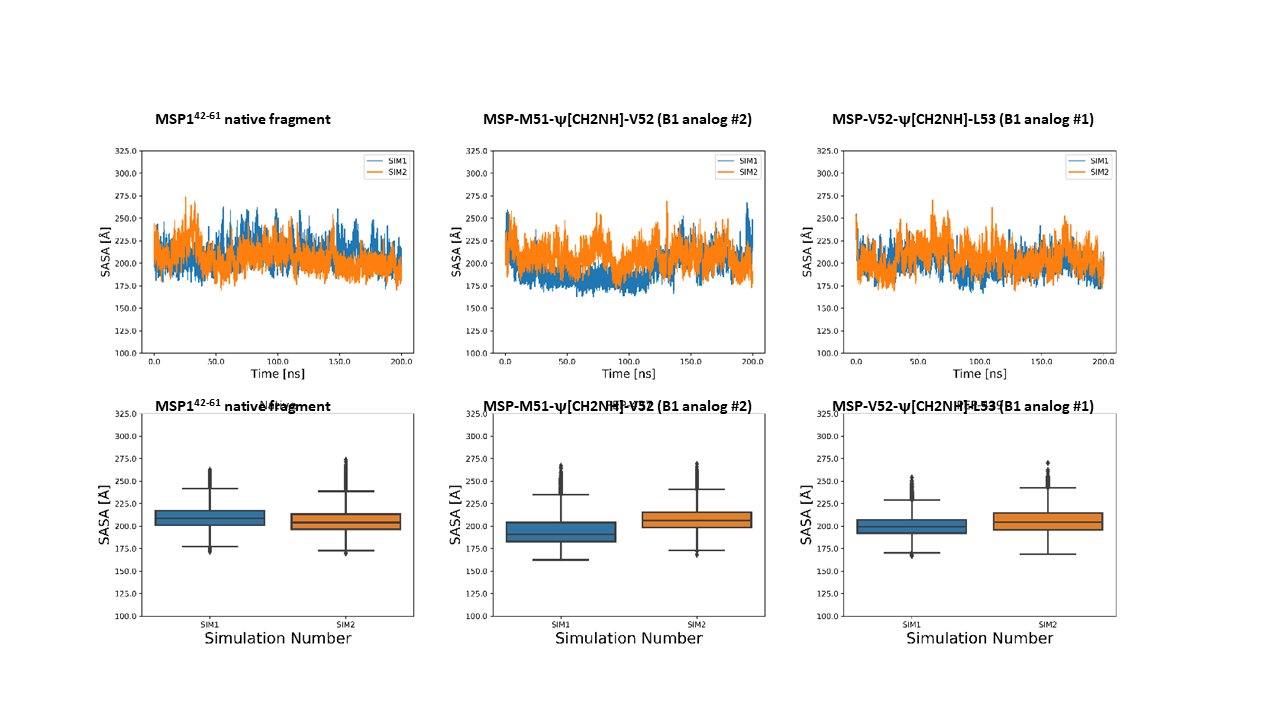

Supplement: Supplementary file 1 [file molecules-28-02527-s001.zip › Figure S5.TIF]

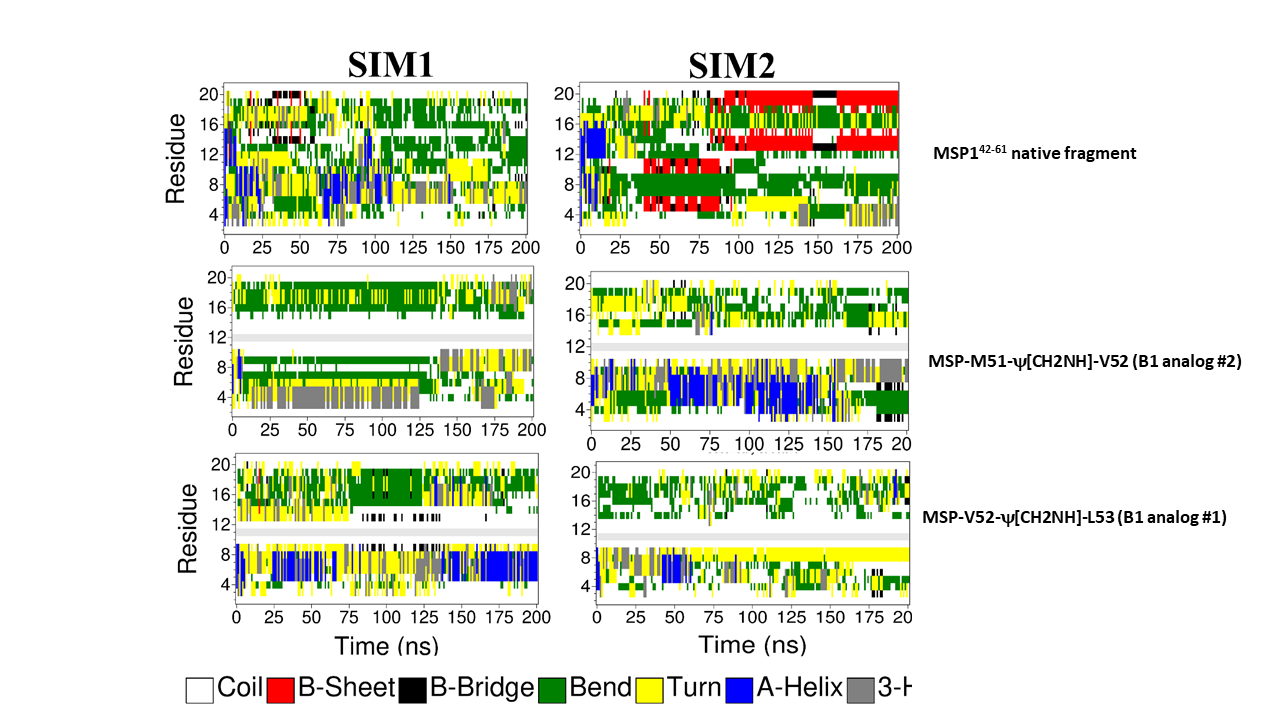

Supplement: Supplementary file 1 [file molecules-28-02527-s001.zip › Figure S6.TIF]

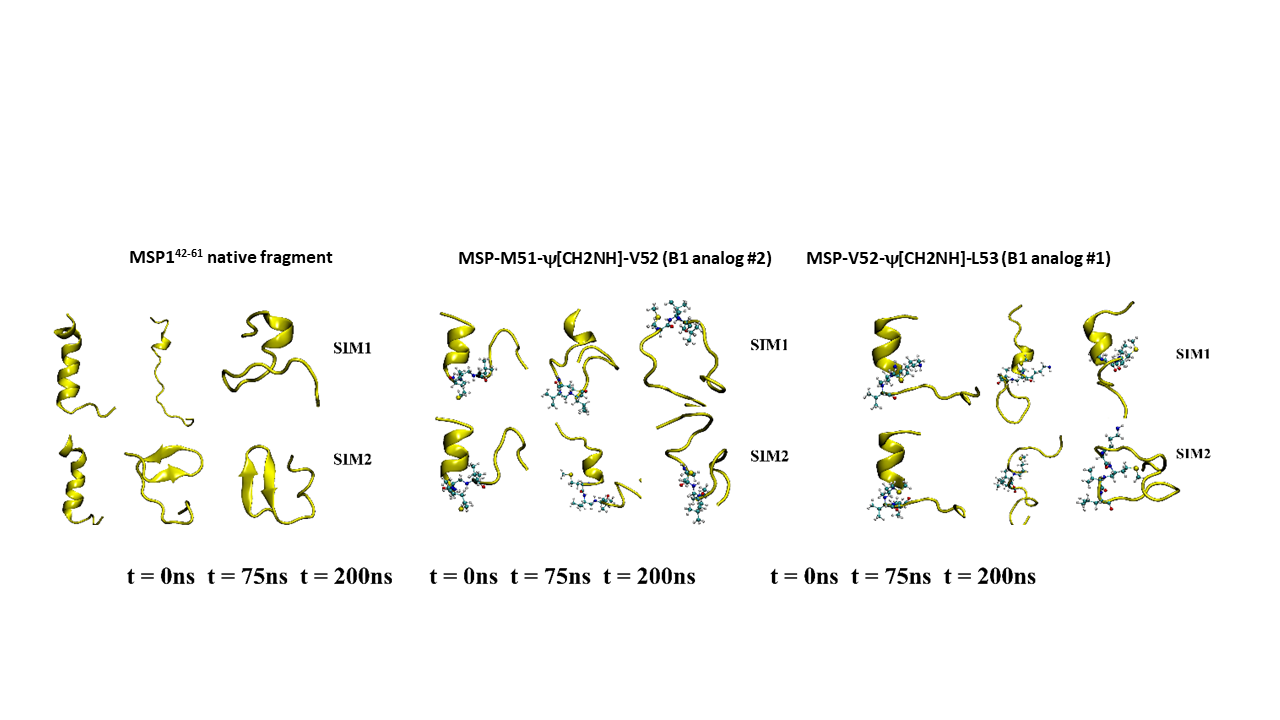

Supplement: Supplementary file 1 [file molecules-28-02527-s001.zip › Figure S7.TIF]
